# Progressive Microbial Community Networks with Incremental Organic Loading Rates Underlie Higher Anaerobic Digestion Performance

**DOI:** 10.1128/mSystems.00357-19

**Published:** 2020-01-07

**Authors:** Linwei Wu, Xiaoyu Shan, Si Chen, Qiuting Zhang, Qi Qi, Ziyan Qin, Huaqun Yin, Jizhong Zhou, Qiang He, Yunfeng Yang

**Affiliations:** a Institute for Environmental Genomics, University of Oklahoma, Norman, Oklahoma, USA; b Department of Microbiology and Plant Biology, University of Oklahoma, Norman, Oklahoma, USA; c State Key Joint Laboratory of Environment Simulation and Pollution Control, School of Environment, Tsinghua University, Beijing, China; d Department of Civil and Environmental Engineering, The University of Tennessee, Knoxville, Tennessee, USA; e School of Minerals Processing and Bioengineering, Central South University, Changsha, China; f Earth Sciences Division, Lawrence Berkeley National Laboratory, Berkeley, California, USA; g Institute for a Secure and Sustainable Environment, The University of Tennessee, Knoxville, Tennessee, USA; University of Illinois at Urbana-Champaign

**Keywords:** anaerobic digestion, microbial interaction, system performance

## Abstract

Although biotic interactions among members of microbial communities have been conceived to be crucial for community assembly, it remains unclear how changes in environmental conditions affect microbial interaction and consequently system performance. Here, we adopted a random matrix theory-based network analysis to explore microbial interactions in triplicate anaerobic digestion (AD) systems, which is widely applied for organic pollutant treatments. The digesters were operated with incremental organic loading rates (OLRs) from 1.0 g volatile solids (VS)/liter/day to 1.3 g VS/liter/day and then to 1.5 g VS/liter/day, which increased VS removal and methane production proportionally. Higher resource availability led to networks with higher connectivity and shorter harmonic geodesic distance, suggestive of more intense microbial interactions and quicker responses to environmental changes. Strikingly, a number of topological properties of microbial network showed significant (*P* < 0.05) correlation with AD performance (i.e., methane production, biogas production, and VS removal). When controlling for environmental parameters (e.g., total ammonia, pH, and the VS load), node connectivity, especially that of the methanogenic archaeal network, still correlated with AD performance. Last, we identified the *Methanothermus*, *Methanobacterium*, *Chlorobium*, and *Haloarcula* taxa and an unclassified *Thaumarchaeota* taxon as keystone nodes of the network.

**IMPORTANCE** AD is a biological process widely used for effective waste treatment throughout the world. Biotic interactions among microbes are critical to the assembly and functioning of the microbial community, but the response of microbial interactions to environmental changes and their influence on AD performance are still poorly understood. Using well-replicated time series data of 16S rRNA gene amplicons and functional gene arrays, we constructed random matrix theory-based association networks to characterize potential microbial interactions with incremental OLRs. We demonstrated striking linkage between network topological features of methanogenic archaea and AD functioning independent of environmental parameters. As the intricate balance of multiple microbial functional groups is responsible for methane production, our results suggest that microbial interaction may be an important, previously unrecognized mechanism in determining AD performance.

## Abstract (precis)

AD is a biological process widely used for effective waste treatment throughout the world. Biotic interactions among microbes are critical to the assembly and functioning of the microbial community, but the response of microbial interactions to environmental changes and their influence on AD performance are still poorly understood. Using well-replicated time series data of 16S rRNA gene amplicons and functional gene arrays, we constructed random matrix theory-based association networks to characterize potential microbial interactions with incremental OLRs. We demonstrated striking linkage between network topological features of methanogenic archaea and AD functioning independent of environmental parameters. As the intricate balance of multiple microbial functional groups is responsible for methane production, our results suggest that microbial interaction may be an important, previously unrecognized mechanism in determining AD performance.

## INTRODUCTION

Anaerobic digestion (AD) is an efficient industrial process widely applied in organic waste treatment. The conversion of organic matter into biogas relies on cooperation of several microbial trophic groups, i.e., hydrolyzing bacteria, acidogenic bacteria, syntrophic acetogenic bacteria, and methanogenic archaea (1). It is thus essential to understand how microbial functional groups respond to different operating conditions such as variations in organic loading rates (OLRs) and waste composition. Recent development of molecular microbial ecology techniques has enabled great advancements in characterizing microbial community composition in anaerobic digesters, which provide valuable information of core microorganisms as well as population dynamics under different conditions, e.g., maturation of sludge granules and deterioration induced by acidification (2).

Previous studies have shown that AD performance correlates with the relative abundances of functional taxa like methanogenic archaea, acetate-oxidizing syntrophic bacteria, and cellulolytic bacteria (3, 4). However, despite evidence that biotic interactions could contribute more to ecosystem functions than individual populations (5), knowledge regarding potential interactions among microbial community members of AD system remains very limited. Unlike observations in macroecology, a major technical hurdle is that microbial interactions in environmental samples could not be directly observed. To address this, association network algorithms have been developed to infer potential microbial interactions by unveiling strong, nonrandom correlation or cooccurrence patterns between microbial members (6). Positive cooccurrence or correlation between microbial taxa may suggest cross-feeding, coaggregation, cocolonization, and niche overlap, while mutual exclusion or negative correlation could arise from amensalism, prey-predator relationship, competition, and differential niche adaptation (7). Network analyses are advantageous in that not only do they simplify complicated relationships among microbial members but the topological properties of networks could also provide a novel, holistic angle for characterizing communities as emergent properties (8–10). For example, microbial interaction complexity in a semiarid grassland increased with elevated concentration of CO_2_, and network topological properties correlated with soil geochemical parameters, highlighting the important role of microbial interactions in assessing microbial responses to environmental conditions (8). However, most network studies focus on how microbial interaction affects community assembly or responses to environment. To date, there have been few studies to elucidate whether and how microbial interaction affects community functioning.

In this study, we applied random matrix theory (RMT)-based network analysis to bacterial and archaeal organisms to discern putative microbial interactions in anaerobic digesters. The RMT-based network algorithm employed is unique in that it automatically determines the threshold for constructing correlation networks, which is objective and reliable (11, 12). This approach has proved to be a powerful tool in a variety of environments (9, 13, 14). Given the importance of biotic interactions in shaping microbial community (6), our overarching hypothesis is that interactions among microbial members are essential for AD performance. To test this hypothesis, we use 16S time series data collected from AD reactors with different organic loading rates (OLRs) to construct microbial correlation networks and infer putative interactions among community members from network properties. We examine how network topologies change under different resource availability and whether such changes correlate with AD performance, using control digesters fed dairy manure and treatment digesters fed both dairy manure and poultry waste. We expect that higher resource availability under codigestion stimulates various biotic interactions by increasing the metabolic activities of community members (15).

## RESULTS

### AD parameters.

The treatment digesters and the control digesters were operated under the same conditions during days 1 to 44, with dairy manure as the sole substrate. In the treatment digesters, the OLR increased from 1.3 g volatile solids (VS)/liter/day (T 45-76 representing treatment samples during days 45 to 76) to 1.5 g VS/liter/day (T 80-97 representing treatment samples during days 80 to 97) by adding a cosubstrate of poultry waste. Concurrently, there were increases in VS removal, biogas production, and methane production (see Fig. S1 in the supplemental material), indicating that codigestion led to higher AD performance. In contrast, VS removal, biogas production, and methane production in control digesters remained relatively stable throughout the experiment. In addition, we measured other environmental parameters (pH, acetate, total ammonia, and VS load) in both sets of digesters and found that VS load and total ammonia also increased with the elevated OLR (Fig. S1).

10.1128/mSystems.00357-19.1 FIG S1 Operational parameters in the treatment (red) and control (blue) digesters. Mean ± standard deviation (SD) values of AD performance (i.e., biogas production, methane production, and VS removal) and environmental parameters (VS, pH, acetate and total ammonia) are shown by sampling dates. Download FIG S1, TIF file, 1.5 MB. Copyright © 2020 Wu et al. 2020 Wu et al. This content is distributed under the terms of the Creative Commons Attribution 4.0 International license.

### Network topological properties.

Microbial alpha-diversity, represented by the inverse Simpson index (*n*_eff_), ranged from 23.1 to 76.2 across samples but was not significantly different between control and treatment digesters (*P = *0.1) (Fig. S2). The beta-diversity based on Bray-Curtis distance was also calculated to illustrate shifts in microbial community composition. After day 45, there were larger shifts in microbial community composition in treatment digesters (T 45-76 and T 80-97) than in control digesters (C 45-76 [control samples during days 45 to 76] and C 80-97) (Fig. S3), owing to incremental disturbance in treatment digesters. Therefore, we generated five association networks based on the disturbance. We generated an overall network with all 66 samples and four networks with groups of days 45 to 76 and days 80 to 97 in control and treatment digesters to discern the succession of microbial interactions. In all networks, node connectivity fit the power law distribution well (*r*^2^ > 0.74, *P* < 0.01) (Table 1), displaying a scale-free property (16) where a few nodes (potentially keystone species in an ecological network [17]) have proportionally more connections. The average clustering coefficients, which describe the extent to which the nodes tend to cluster together (see Table S1 in the supplemental material), were significantly (*P < *0.05) higher than random expectations. The harmonic geodesic distance, which measures the average shortest path between nodes, was close to the logarithmic value of the network size (the total number of nodes) in each network, showing a small-world property (i.e., the network nodes are closely linked to each other). The small-world structure makes the communications among different members within a system more efficient (18). Networks were modular, since their modularity values were significantly (*P < *0.05) higher than those of the corresponding randomized networks.

**TABLE 1:** Topological properties of the empirical networks versus random networks

Data set	Empirical networks^*a*^								Random networks^*b*^		
	No. of nodes	No. of edges	% Pos edges	*R*^2^ of power law	avgCN	GD	avgCC	Modularity (no. of modules)	GD	avgCC	Modularity
Entire	266	873	91.6	0.79	6.53	5.14	0.231	0.41 (17)	2.92 ± 0.01	0.023 ± 0.003	0.36 ± 0.01
C 45-76	209	356	83.1	0.93	3.37	5.65	0.100	0.65 (17)	4.22 ± 0.08	0.014 ± 0.006	0.54 ± 0.01
T 45-76	223	380	83.9	0.93	3.43	5.55	0.110	0.63 (19)	4.44 ± 0.07	0.012 ± 0.006	0.54 ± 0.01
C 80-97	217	515	88.3	0.86	4.75	5.17	0.154	0.52 (18)	3.60 ± 0.03	0.019 ± 0.005	0.45 ± 0.01
T 80-97	250	704	90.1	0.74	5.63	5.11	0.190	0.51 (17)	3.38 ± 0.01	0.022 ± 0.005	0.39 ± 0.01

a % Pos edges, percent positive edges; avgCN, average connectivity; GD, harmonic geodesic distance; avgCC, average clustering coefficient.

b The mean values ± standard deviations from 100 random networks are shown.

10.1128/mSystems.00357-19.2 FIG S2 Microbial alpha-diversity represented by the inverse Simpson index (*n*_eff_) across different digesters. The alpha-diversity of control digesters (C1, C2, and C3) and treatment digesters (T1, T2, and T3) is not significantly different according to the ADONIS test (*P* = 0.1). The *n*_eff_ ranged from 23.1 to 76.2. Download FIG S2, TIF file, 0.3 MB. Copyright © 2020 Wu et al. 2020 Wu et al. This content is distributed under the terms of the Creative Commons Attribution 4.0 International license.

10.1128/mSystems.00357-19.3 FIG S3 Shift of microbial community structure compared to those of C45 and T45, i.e., samples from control digesters on day 45 and from treatment digesters on day 45. There are larger shifts in microbial community composition in treatment digesters than control digesters, as indicated by larger dissimilarity of community structure quantified by beta-diversity analysis using Bray-Curtis distance. Download FIG S3, TIF file, 0.4 MB. Copyright © 2020 Wu et al. 2020 Wu et al. This content is distributed under the terms of the Creative Commons Attribution 4.0 International license.

10.1128/mSystems.00357-19.5 TABLE S1 Network topological properties used in this study. Download Table S1, DOCX file, 0.02 MB. Copyright © 2020 Wu et al. 2020 Wu et al. This content is distributed under the terms of the Creative Commons Attribution 4.0 International license.

The average connectivity and average clustering coefficient were higher in treatment digester networks than control digester networks, and the harmonic geodesic distance of treatment networks was shorter than control networks (Table 1). These results suggest that the overall interaction intensity (i.e., the extent to which members of the community are linked) was higher in treatment digesters. Among the four networks, the highest average connectivity and clustering coefficient and the shortest harmonic geodesic distance were observed in T 80-97, suggesting these networks properties were related to the elevated resource availability. The network edges were predominantly positive (>83% of total edges), with slightly higher positive percentages in the treatment networks.

### Linkages between network topology and AD parameters.

**FIG 1:**
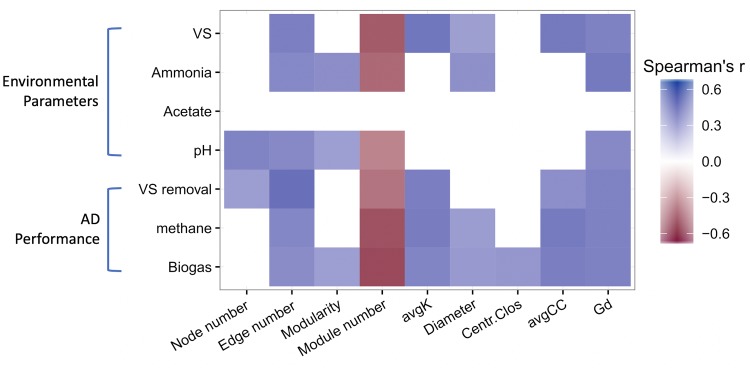
Spearman correlation between network topological properties and AD parameters. The significance of correlations is determined as *P* < 0.05, and only significant correlations are visualized in the figure. avgK, the average degree of network; avgCC, average clustering coefficient; Centr.Clos, centralization of closeness; Gd, harmonic geodesic distance.

We examined correlations between network topological properties and AD parameters (Fig. 1). The edge number and harmonic geodesic distance positively correlated with the VS load, total ammonia, pH value, VS removal, methane production, and biogas production (*r *> 0.38, *P < *0.05), while the module number negatively correlated with those AD parameters (*r* < −0.36, *P < *0.05) (Fig. 1). The average connectivity and average clustering coefficient positively correlated with VS load, VS removal, methane production, and biogas production (*r *> 0.36, *P < *0.05), suggesting that denser network structures were related to higher VS removal and biogas production.

To further analyze the relationship between network topology and AD parameters, we calculated the gene significance (GS) as the square of Pearson correlation coefficient (*r*^2^) of operational taxonomic unit (OTU) abundance with corresponding AD parameters in the overall network as previously described (16, 19). The correlations between the connectivity of nodes and AD parameters were examined by Mantel tests, which showed that nodes’ connectivity significantly (*P* = 0.001) correlated with the GS of environmental parameters (represented by VS, total ammonia, acetate, and pH value), as well as with the GS of AD performances of VS removal, biogas production, and methane production (Table 2; see Materials and Methods for details). However, partial Mantel test showed that the correlation between nodes’ connectivity and the GS of AD performances was not significant when controlling the effects of environmental parameters. In sharp contrast, the connectivity of archaea significantly correlated with the GS of environmental parameters (*r *= 0.46, *P = *0.001) and with that of AD performances (*r *= 0.48, *P* = 0.001) even when environmental parameters were controlled (*r *= 0.21, *P = *0.001). Nodes from archaeal classes *Methanobacteria*, *Methanococci*, *Methanomicrobia*, and *Thermoprotei* showed similar results, as there were significant correlations (*P* < 0.05) with environmental parameters and AD performances. When environmental parameters were controlled in partial Mantel test, there was still significant correlation (*r *= 0.55, *P* = 0.007) between the connectivity of *Methanococci* nodes and the GS of AD performances. In contrast, the correlation between the connectivity of bacteria and the GS of AD performances was not significant (*r* = −0.02, *P* = 0.78) when environmental parameters were controlled. Notably, *Bacteroidia* was the only bacterial taxon whose node connectivity correlated (*r *= 0.21, *P* = 0.023) with AD performances independent of the effects of environmental parameters.

**TABLE 2:** Mantel tests on connectivity and the gene significance of environmental parameters and AD performance in the overall network^*a*^

Category	GS of ENV^*b*^		GS of AD performance^*c*^		GS of AD performance (ENV controlled)^*d*^	
	*r_M_*	*P*^*e*^	*r_M_*	*P*	*r_M_*	*P*
All	0.195	0.001*	0.181	0.001*	0.022	0.147
*Archaea*	0.455	0.001*	0.475	0.001*	0.213	0.001*
*Methanobacteria*	0.515	0.002*	0.514	0.003*	0.281	0.050*
*Methanococci*	0.468	0.032	0.666	0.003*	0.548	0.007*
*Methanomicrobia*	0.376	0.001*	0.378	0.001*	0.113	0.033*
*Thermococci*	0.380	0.013*	0.407	0.001*	0.166	0.082
*Thermoplasmata*	−0.196	0.834	0.098	0.352	0.448	0.046*
*Thermoprotei*	0.513	0.001*	0.609	0.001*	0.418	0.001*
*Archaeoglobi*	−0.001	0.392	0.211	0.207	0.289	0.132
*Bacteria*	0.123	0.001*	0.101	0.001*	−0.019	0.780
*Acidimicrobiia*	−0.183	0.880	−0.158	1.000	−0.027	0.358
*Actinobacteria*	0.405	0.001*	0.343	0.002*	−0.018	0.546
*Anaerolineae*	0.148	0.113	0.087	0.225	−0.054	0.583
*Bacteroidia*	0.514	0.001*	0.544	0.001*	0.213	0.027*
*Bacilli*	0.356	0.006*	0.233	0.028*	−0.136	0.963
*Clostridia*	0.092	0.013*	0.080	0.006*	−0.002	0.504
*Erysipelotrichia*	0.411	0.104	0.482	0.059	0.276	0.121
*Alphaproteobacteria*	0.105	0.280	0.184	0.106	0.156	0.157
*Betaproteobacteria*	0.079	0.329	−0.061	0.537	−0.212	0.869
*Gammaproteobacteria*	−0.154	0.861	−0.159	0.847	−0.044	0.528
Deltaproteobacteria	−0.164	0.937	−0.090	0.737	0.152	0.109

a The gene significance (GS) of environmental parameters and AD performance is shown. *r_M_*, the correlation coefficient based on Mantel test.

b Environmental parameters (ENV) include total ammonia, acetate, pH, and VS load.

c AD performance includes biogas production, methane production, and VS removal.

d Partial Mantel test was applied to determine correlation between connectivity and GS of AD performance, in which GS of environmental parameters were controlled.

e Asterisks represents significance of Mantel test with *P* < 0.05.

### Associations between microbial taxa.

**FIG 2:**
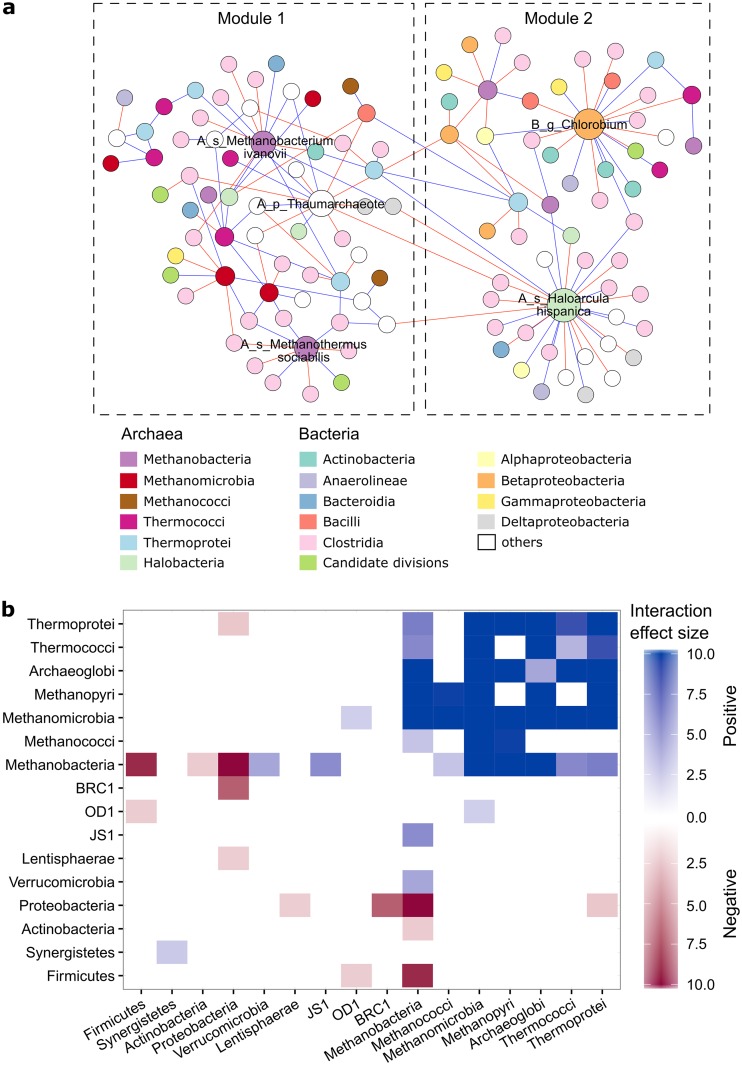
Interactions between microbial taxa revealed by the association network. (a) The two largest modules in the overall network constructed from all 66 samples. Nodes are colored according to OTU taxa. Labeled nodes are the hubs identified as module hubs. Node labels stand for the lowest taxonomic rank (A_ and B_ represent *Archaea* and *Bacteria*, respectively; p_, g_, and s_ represent phylum, genus, and species, respectively) of OTUs, and the node size is proportional to its degree. Blue edges represent positive associations, while red edges represent negative associations. (b) Interaction effect size between microbial taxa. The interaction effect size between any two groups was calculated as the difference of observed and random networks divided by the standard deviation. The heatmap shows only interactions with an effect size larger than 2.

The overall network contained 873 edges among 266 microorganisms, of which 343 edges were global and 530 edges were group specific (see Materials and Methods for details). Most edges (707/873) were within archaea, with much smaller numbers of edges between archaea and bacteria (106/873) and within bacteria (60/873). A total of 86 edges between archaea and bacteria were global, suggesting that they were conserved under different OLRs. Figure 2a showed the interactions among nodes of the two largest modules within the overall network. Archaeal nodes were associated with a variety of bacterial taxa, especially with *Clostridia* nodes. In the first module, all the three module hubs were archaeal nodes, belonging to Methanobacterium ivanovii, Methanothermus sociabilis, and an uncultured *Thaumarchaeote*. The two hubs for the second module belonged to a bacterial genus *Chlorobium* and an archaeal species, Haloarcula hispanica.

To determine whether the associations between microorganisms were significant, we compared empirical edges with random expectations by null model test. Significant associations (i.e., interaction effect size of >2) are shown in Fig. 2b. The number of positive edges between methanogens and uncultivated bacterial phyla candidate divisions OD1 and JS1 was significantly higher than random values. Significant, positive associations between archaeal organisms were also evident, as shown by edges between *Archaeoglobi* and *Methanobacteria* and between *Thermoprotei* and *Methanomicrobia*. Within bacteria, *Proteobacteria* significantly and negatively correlated with *Lentisphaerae* and candidate division BRC1. Closer examination showed that *Betaproteobacteria* significantly and negatively correlated with both *Bacilli* and *Clostridia*.

## DISCUSSION

In this study, we observed changes in microbial association networks with different resource availability. Consistent with our expectation, the network topology properties suggested increased microbial interaction intensity with higher resource availability. The size of the network (i.e., the number of nodes and edges) increased with higher resource availability (Table 1), which could be interpreted as an increased efficiency of utilization of matter and energy (20). With the decrease in harmonic geodesic distance and the increase in the clustering coefficient (Table 1), the network nodes became closer to each other with higher resource availability levels. Changes in key network topological properties (i.e., average connectivity and average clustering coefficient) were higher in treatment digesters than in control digesters. Together with larger shifts in community composition revealed by beta-diversity analysis, the results implied that the elevated resource availability by codigestion resulted in faster community structure succession and intensification of microbial interactions in anaerobic digesters.

The most striking finding of this study is significant linkages between AD performance and network topological properties (Fig. 1 and Table 2). AD performance largely depends on microbes being functionally active to consecutive phases of hydrolysis, acidogenesis, and methanogenesis and microbes being functionally interactive between hydrogenotrophic methanogens and syntrophs to bridge the thermodynamic gaps (21). A negative correlation was observed between the number of modules and AD performance (Fig. 1). As previous studies have interpreted modules as niches (22, 23), decrease in the number of modules was suggestive of niche adaptation, which might enhance VS removal and methane production. Meanwhile, the positive correlation between the degree of connectivity and AD performance suggests that microbial interactions contributed to AD performance. Possible mechanisms include increased metabolic activity/growth from increased resource concentration and/or the introduction of relevant community members from the additional feed stock.

By differentiating bacterial and archaeal nodes, we showed that connectivity of archaeal nodes had significant correlation with AD performance independent of environmental parameters (Table 2). Microorganisms associated with in AD performance are comprised of both bacteria and archaea, of which archaea usually accounts for only ∼5% of total genomic sequence reads (24). However, other studies have shown that archaea can account for as much as 30% of total transcripts, suggesting the disproportionate importance of archaea in terms of activities and function (25, 26). In addition, the stability of our treatment digesters was linked to the robust archaeal microbial community, reinforcing the crucial role of archaea for AD performance (27). Indeed, archaea exhibited stronger associations with biogas production than bacteria, which could be attributed to the fact that the terminal phase of methanogenesis is exclusively carried out by archaea. Therefore, the connectivity of archaeal nodes was strongly related to AD performance.

Identifying keystone species is important because loss of the species that has the highest number of edges within the network should have the greatest effects on community structure (28). Five module hubs were identified in the two largest modules (Fig. 2a). Among the five hubs, the genus *Methanobacterium* and *Methanothermus* have been well documented for oxidizing H_2_ to produce methane (29). The members of the genus *Haloarcula* could transform sugars to acids during acidogenesis (29). The genus *Chlorobium* affiliated with the phylum *Cholorobi* is known as green sulfur bacteria that employ reverse citric acid cycle to reduce CO_2_ with H_2_. Therefore, it could potentially compete with methanogens (30). Another module hub is a member in the novel archaeal phylum *Thaumarchaeota*, which was previously classified to be mesophilic *Crenarchaeotes*. *Thaumarchaeota* is abundant in anaerobic digesters, accounting for 4.8% of all archaeal sequence reads (31). It might possess heterotrophic metabolism and hence compete with heterotrophic bacteria (32). Here, identification of *Thaumarchaeota* as module hubs raises the possibility that it might be a keystone generalist in anaerobic digesters (33). Notably, a variety of bacteria, predominantly *Clostridia*, were associated with module hubs. Members of *Clostridia* have been well documented as hydrolytic and fermentative bacteria and were recently identified as core taxa in anaerobic digesters (34, 35).

The effect size analysis of association networks revealed specific patterns of cooccurrence between microbial taxa (Fig. 2b). Most of those positively correlated archaea are known as hydrogenotrophic methanogens; hence, significant, positive correlation might suggest niche overlap among those taxa. Uncultivated bacterial phyla candidate divisions OD1 and JS1 positively correlated with methanogens (Fig. 2b). Recent metabolic pathway reconstruction of OD1 revealed a potential for fermenting small organic compounds (36), while genomic evidence for JS1 showed a saccharolytic, fermentative lifestyle amenable for cellulose degradation and hydrogen production (37). Therefore, the correlation between JS1 and methanogens in our study could suggest a syntrophic relationship.

Correlation structure in microbial relative abundances was used to infer underlying ecological interactions. As is well-known, the nature of these amplicon-based data, such as compositionality and sparsity, could bring in spurious indirect correlations (17, 38). It is suggested that the compositional effects should be considered for communities with lower effective numbers of species (*n*_eff_ < 13), but the effects are generally insignificant for highly diverse communities (39). The *n*_eff_ values of our AD microbiomes were larger than 23, suggesting a minor effect of the compositional data structure. We accounted for some of the sparsity by filtering out rare OTUs prior to network construction. The cutoff value of correlation coefficient (i.e., 0.86) was relatively high, which could also reduce the rates of false-positive results. Nevertheless, we could not exclude the possibility that the edges we discovered did not represent direct species-species associations. A recent study reported that although association network analyses could predict the high-level network topology, there was a huge mismatch between species associations and experimentally determined species interactions (40).

In summary, this paper reveals significant linkages between microbial community network properties and AD performance, which suggests that microbial interaction could be essential for AD processes. Increases in edge number and clustering coefficient and decreases in harmonic geodesic distance were observed in associate networks with elevated resource availability. Given the potential role of microbial interactions in AD, these results suggest that elevated resource availability leads to intensification of interactions, which may facilitate the observed increases in AD performance. As network topological properties, particularly for archaea, significantly correlate with AD performance, topological properties such as degree of connectivity and number of modules could potentially serve as predictors for AD performance. The identification of *Methanobacterium*, *Methanothermus*, and unclassified *Thaumarchaeota* species as keystone nodes is exciting, which offers valuable insights into the development of functional community and highlights the future knowledge gap-filling that will be addressed. Those advancements may not be easy, but they are possible. If undertaken, further studies could take much of the uncertainty out of AD stability and could provide environmental engineers novel intellectual ideas to upgrade AD processes.

## MATERIALS AND METHODS

### Anaerobic digester operation and sample collection.

Two sets of triplicate, lab-scale continuous anaerobic digesters (i.e., the treatment digesters [indicated by “T”] and control digesters [indicated by “C”]) with a working volume of 3.6 liters and a hydraulic retention time of 20 days were established. All of the digesters were initiated with inoculum from an operating laboratory dairy manure anaerobic digester and with dilute dairy waste as the sole substrate. All digesters were operated at 35°C and fed at 4-h intervals.

During days 1 to 44, both sets of digesters were run with a constant organic loading rate (OLR) of 1.0 g volatile solids (VS)/liter/day with dairy waste as the sole substrate, resulting in stable operation with steady pH, methane yield, and volatile fatty acid (VFA) level. Then, the OLR in the treatment digesters was raised to 1.3 g VS/liter/day with the addition of poultry waste on day 45 by adding poultry waste as the substrate of codigestion and further to 1.5 g VS/liter/day during days 80 to 97, while the OLR of the control digesters remained unchanged. Digestate samples, i.e., materials exiting anaerobic digesters, were taken on days 45, 62, 66, 69, 73, 76, 80, 83, 87, 90, and 97. In total, we collected 66 samples.

### Chemical analyses.

The biogas production of anaerobic digesters was quantified by the water displacement method (41). Methane content in biogas was measured by a Hewlett Packard 5890 series II gas chromatograph (Agilent Technologies, Santa Clara, CA, USA) with a thermal conductivity detector (TCD) and a Supelco packing column (60/80 Carbonxen-1000; Sigma-Aldrich, St. Louis, MO, USA). Biogas production and methane production were normalized to standard temperature and pressure (i.e., 273.15 K and 101.33 kPa). Argon was used as the carrier gas at a ﬂow rate of 5 ml min^−1^ under the following temperature scheme: 125°C in the oven, 150°C at the injection port, and 170°C in the detector. Acetate was determined with an Agilent 1200 series high-performance liquid chromatograph (Agilent Technologies, Santa Clara, CA, USA) equipped with a Bio-Rad Aminex HPX-87H ion exclusion column (Bio-Rad, Hercules, CA, USA) heated to 60°C with 0.005 N sulfuric acid as the eluent. Ammonia and VS were measured by the standard method (42). In short, ammonia was measured by the “4500NH_3_ D”’ method with an Orion 9512 ammonia ion-selective electrode (Orion Research Inc., Beverly, MA, USA). VS was measured with the “2540 E” method (42) as the weight loss on ignition at 550°C in a muffle furnace to estimate the amount of organic matter. Amounts of waste feedstock were determined to maintain the desired OLRs of digesters with the quantification of the VS load of dairy and poultry. VS removal was calculated on the basis of the difference between the input VS amount and the VS amount measured in the digesters.

### DNA extraction, 16S rRNA gene amplicon sequencing, and DNA hybridization with GeoChip 5.0.

DNA was extracted from digestate samples as described previously (43). In short, digestate samples were suspended in 630 ml DNA extraction buffer prior to the sequential treatment with 60 ml of a lysozyme mixture (37°C, 60 min), 60 ml of a protease mixture (37°C, 30 min), and 80 ml of 20% sodium dodecyl sulfate (37°C, 90 min). The sample suspension was then extracted with phenol-chloroform-isoamyl alcohol (25:24:1) at 65°C for 20 min, and the supernatant was extracted with chloroform-isoamyl alcohol (24:1). DNA extract was mixed with 0.6 volume of isopropanol and stored at 4°C overnight. DNA was precipitated by centrifugation and then washed with 70% cold ethanol before drying and resuspended in nuclease-free water. A NanoDrop spectrophotometer (NanoDrop Technologies Inc., Wilmington, DE, USA) was used to quantify DNA concentration and purity.

Bacterial community compositions were examined by sequencing with a MiSeq instrument (Illumina, San Diego, CA, USA). The V4 region of microbial 16S rRNA genes was amplified by primer pairs 515F (50-GTG CCA GCM GCC GCG GTA A-30) and 806R (50-GGA CTA CHV GGG TWT CTA AT-30). PCR was performed as follows: 94°C for 1 min; 30 cycles, with 1 cycle consisting of 94°C for 20 s, 53°C for 25 s, and 68°C for 45 s; and a final extension step of 68°C for 10 min. The AccuPrime high-fidelity *Taq* polymerase (Invitrogen, Grand Island, NY, USA) was used in PCR. PCR products were pooled and purified using the QIAquick gel extraction kit (Qiagen, Valencia, CA, USA), and amplicon sequencing with MiSeq was performed at the Institute for Environmental Genomics, University of Oklahoma, Norman, OK, USA.

The primer sequences were trimmed from the paired-end sequences. FLASH was adopted to merge the sequences after trimming (44). Merged sequences were used to generate operational taxonomic units (OTUs) by UPARSE at the sequence similarity threshold of 97% (45). Taxonomy assignment was performed with a confidence cutoff of 50% using the RDP classifier. The OTU matrices were rarefied to 11,558 sequences per sample.

Archaeal community compositions were profiled by GeoChip, which is a microarray-based tool for profiling of microbial communities in a variety of environments (46–49). The latest version of functional gene array, GeoChip 5.0 (60 K), was used. It contains more than 57,000 oligonucleotide probes, covering more than 144,000 gene sequences from 393 gene families related to nitrogen (N), carbon (C), sulfur (S), and phosphorus (P) cycling, metal resistance, organic remediation, among many processes (15). In this study, 500 ng of purified DNA was labeled with Cy 3 and then resuspended in hybridization solution. DNA hybridization with GeoChip was conducted at 67°C in a hybridization oven for 24 h (Agilent Technologies, Santa Clara, CA, USA). After hybridization, the slides were washed with Agilent Wash Buffer I for 5 min and then with Buffer II for 1 min. Slides were scanned with a NimbleGen MS200 microarray scanner (Roche NimbleGen Inc., Madison, WI, USA). Images were extracted, and signals were quantified by the Agilent Feature Extraction program. Raw data were processed by the GeoChip Microarray Data Manager pipeline (http://ieg.ou.edu/microarray/) as previously described (50). Poor-quality spots or those with a signal-to-noise ratio of less than 2 were removed. Signals were normalized within each sample and across all samples, and spots detected in only one sample were removed. The functional genes derived from archaea were selected. For each archaeal organism, the mean signal intensity of functional genes derived from the organism was averaged to indicate its relative abundance. The dynamics of bacterial and archaeal community compositions are shown in Fig. S4.

### Network analyses.

To minimize the possibility of false-positive results, we considered only bacteria and archaea detected in more than 9 samples out of 66 samples. Spearman correlation was used to generate association networks. The nodes in the networks represent microorganisms, and the edges connecting these nodes represent correlations between microorganisms in relative abundances. All *P* values were adjusted for multiple testing using the Benjamini and Hochberg false-discovery rate (FDR) controlling procedure (51). We constructed the overall association network using all samples and Spearman correlation coefficients and a cutoff of 0.001 for FDR-adjusted *P* values. The cutoff for correlation coefficients was then determined as 0.86 through random matrix theory-based methods (52).

Subnetworks were reconstructed for control and treatment samples at each OLR level from the overall network. Samples were divided into four groups: samples from control digesters during days 45 to 76 (C 45-76), samples from treatment digesters during days 45 to 76 (T 45-76), samples from control digesters during days 80 to 97 (C 80-97), and samples from treatment digesters during days 80 to 97 (T 80-97). The impact of each sample group on the edges (Spearman correlations) within the overall network was assessed by dividing the omission score (OS) (Spearman correlation coefficient without these samples) by the absolute original Spearman score (Spearman correlation coefficient) (5). Taking the group size into consideration, the OS of random, same-size sample sets was repeatedly computed 500 times. Nonparametric *P* values were calculated as the number of times that random OSs were smaller than the sample group OS, divided by 500 (the total number of random OSs). Edges were determined to be group specific when OSs were less than the absolute original scores and the *P* values were below 0.05. That is, these edges in the overall network were mainly contributed by the samples in the group. The group-specific edges were then used to construct the subnetwork for each group.

Network properties were calculated with the *igraph* package in R. A set of topological properties was analyzed, as shown in Table S1 in the supplemental material. The topological properties include the number of nodes, the number of edges, average degree (avgK), centralization of degree (CD), average cluster coefficient (avgCC), harmonic geodesic distance (GD), centralization of betweenness (CB), the number of modules, and modularity.

### Statistical analyses.

We compared association networks with random networks. For each network identified, a total of 100 randomly rewired networks were generated with the numbers of nodes and edges unchanged but the positions of all edges randomly rewired. The topological properties were compared between the original network and random networks. To analyze whether the identified interactions (i.e., edges) between phylogenetic groups were significant, the effect size of microbial edges between any two microbial phyla was calculated, which was the difference of the observed edge number between the two groups in the overall network and the mean of edge numbers between the two groups from the 100 random networks, divided by the standard deviation of edge numbers from random networks. An effect size larger than 2 indicates that the phyla interacted more intensely than would be predicted for random expectations.

Two statistical approaches were used to examine the linkage between network properties and AD performances. First, Spearman correlations between network topological properties and AD performances (i.e., VS removal, biogas production, and methane production) were tested. We generated subnetworks for each sample from the overall network by preserving organisms presented in the sample using *subgraph* functions in *igraph* package (53). Network topological properties were calculated for each network, and their correlations with AD performance were then tested. Second, Mantel tests and partial Mantel tests were performed to analyze the correlation between network connectivity and the gene significance (GS) of AD performances, with environmental parameters (i.e., total ammonia, acetate, pH, and the VS load) controlled in the partial Mantel tests. GS was calculated as the square of Pearson correlation coefficient (*r*^2^) of OTU abundance profile with environmental traits as previously described (16). The performance-based GS matrix and the environment-based GS matrix were calculated, which captured the relative significance of OTUs relating to AD performance and environmental parameters (total ammonia, acetate, pH, and the VS load), respectively. Based on the two GS matrices, two distance matrices were then derived to show differences in performance-based GS and environment-based GS among OTUs. A distance matrix among OTUs’ connectivity was also calculated, and its correlations with GS distances were then examined by Mantel or partial Mantel test. All the statistical analyses described above were performed in R (54).

### Data availability.

Sequence data are accessible in the GenBank database under accession number SRP070491. GeoChip data are accessible in the GenBank database under accession number GSE93419.

10.1128/mSystems.00357-19.4 FIG S4 Community composition dynamics across different digesters. Archaeal (a) and bacterial (b) taxa are separately shown as bar plots. Download FIG S4, TIF file, 2.0 MB. Copyright © 2020 Wu et al. 2020 Wu et al. This content is distributed under the terms of the Creative Commons Attribution 4.0 International license.
