# Supplementary figures and images for: Progressive Microbial Community Networks with Incremental Organic Loading Rates Underlie Higher Anaerobic Digestion Performance
Source: mSystems. 2020 Jan 7;5(1):e00357-19. doi: 10.1128/mSystems.00357-19 (PMC6946792; doi:10.1128/mSystems.00357-19)

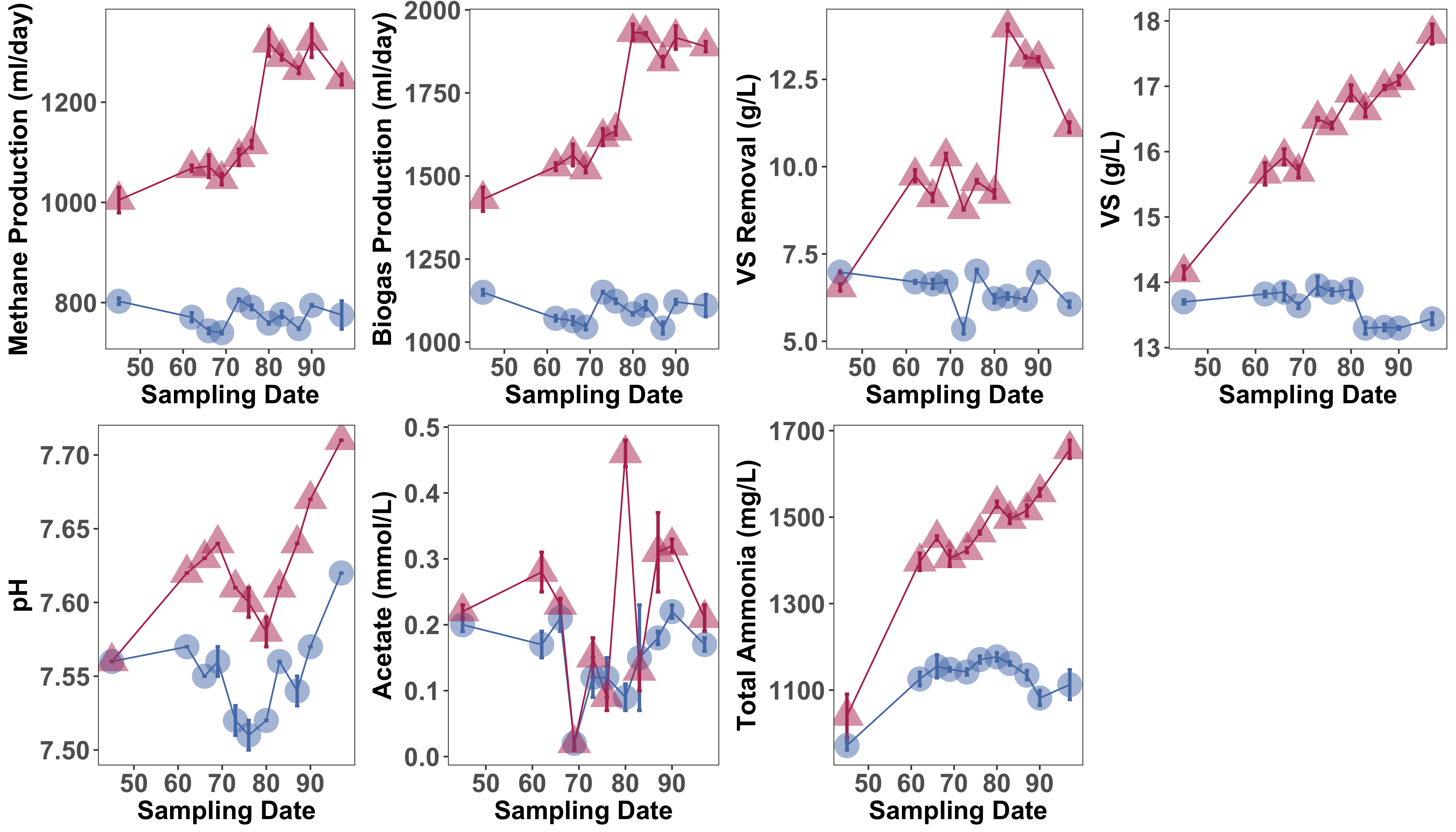

Supplement: FIG S1 [file mSystems.00357-19-sf001.tif]

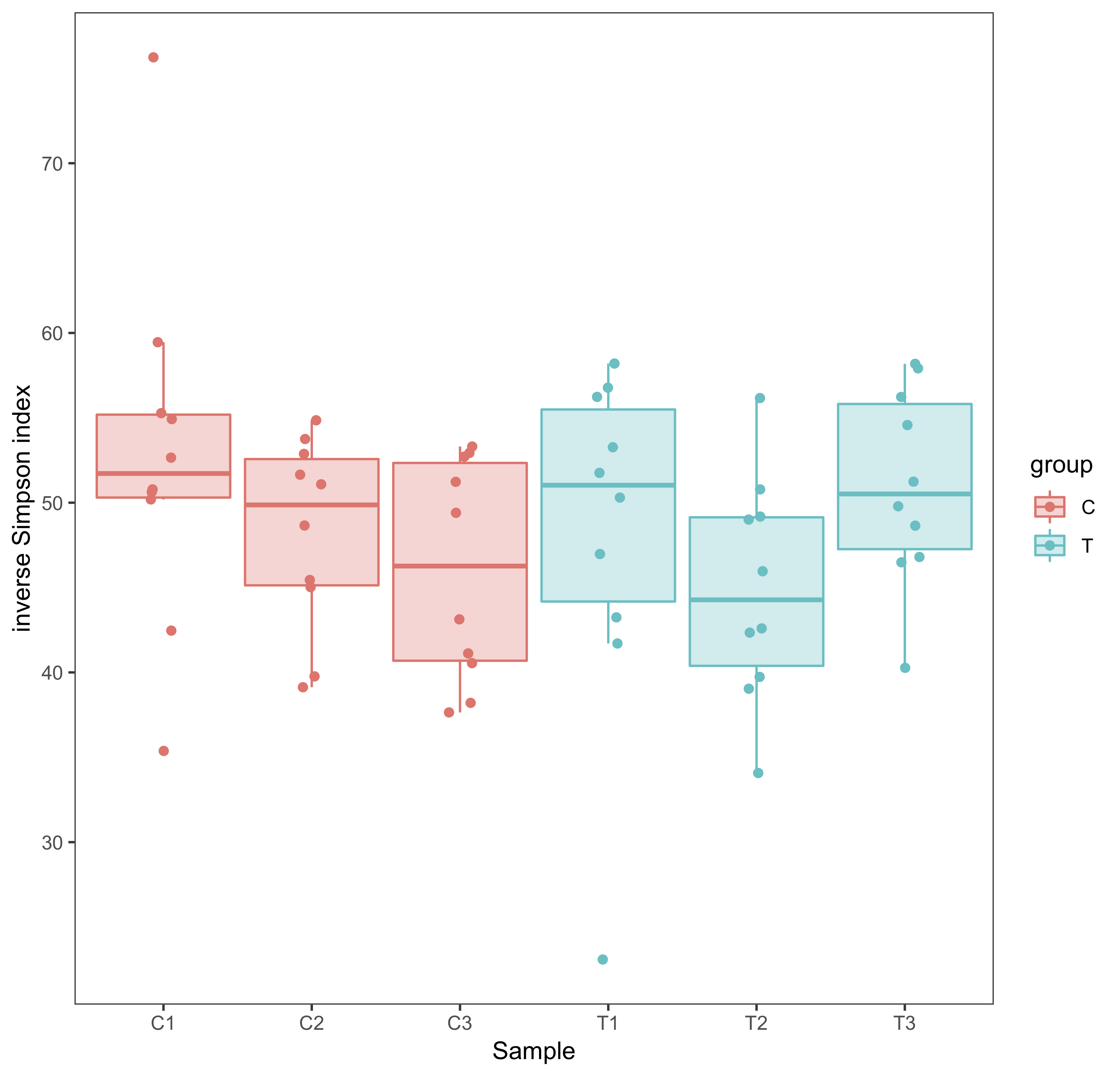

Supplement: FIG S2 [file mSystems.00357-19-sf002.tif]

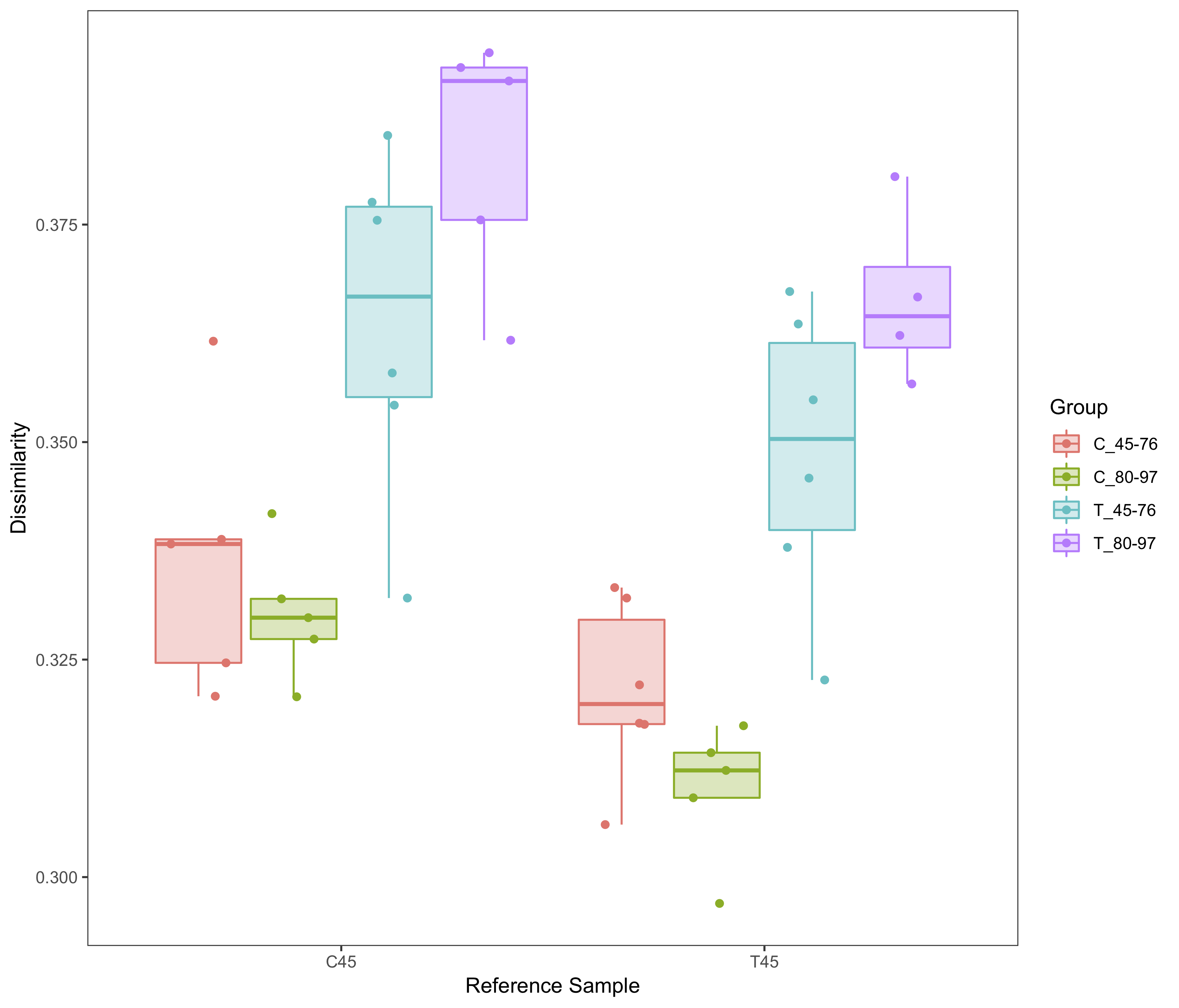

Supplement: FIG S3 [file mSystems.00357-19-sf003.tif]

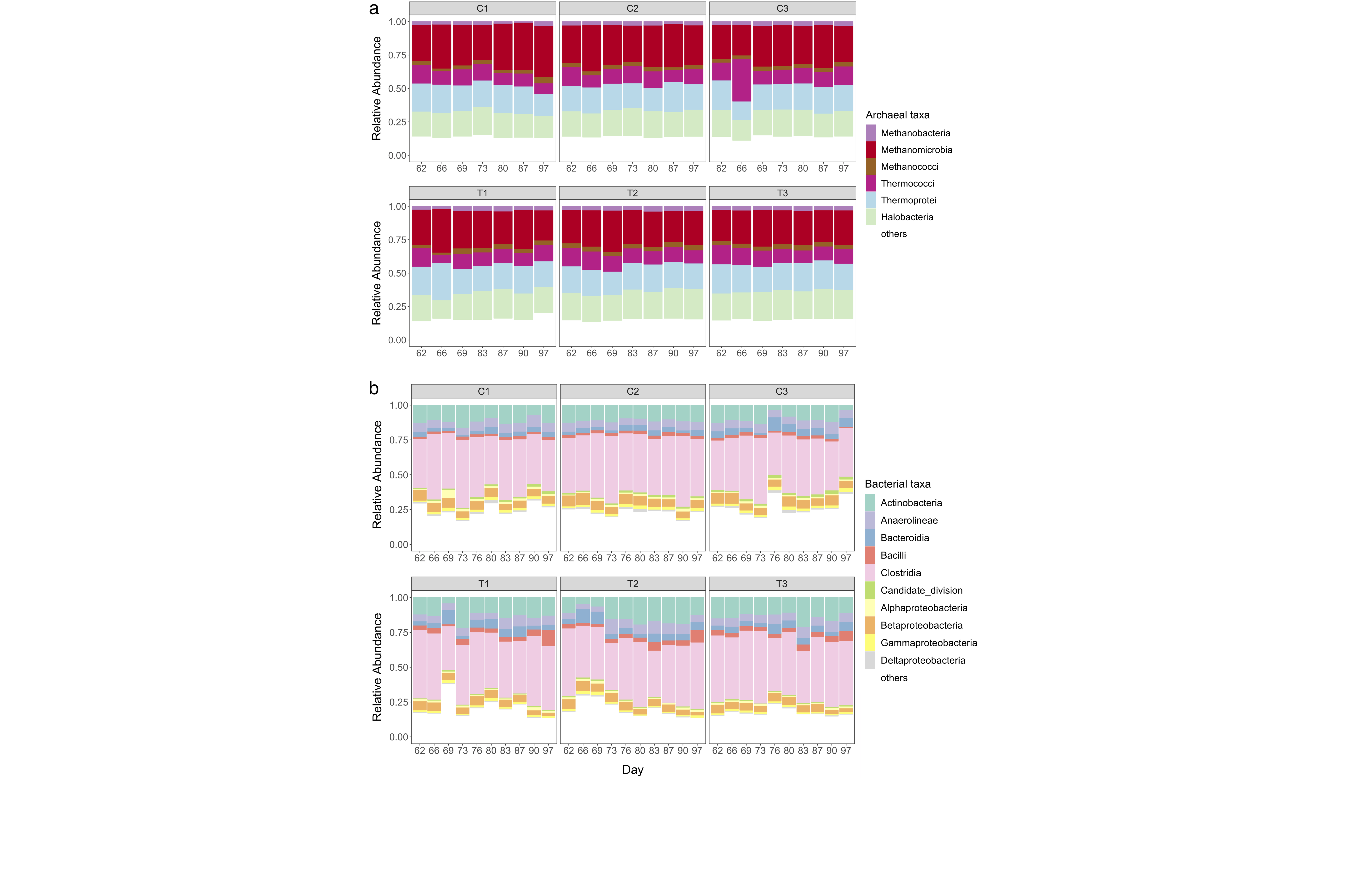

Supplement: FIG S4 [file mSystems.00357-19-sf004.tif]
