# Supplementary material for: Progressive Microbial Community Networks with Incremental Organic Loading Rates Underlie Higher Anaerobic Digestion Performance
Source: mSystems. 2020 Jan 7;5(1):e00357-19. doi: 10.1128/mSystems.00357-19 (PMC6946792; doi:10.1128/mSystems.00357-19)
Supplement: TABLE S1 [file mSystems.00357-19-st001.docx]

**Table S1** The network topological properties used in this study.

| **Features** | **Formula** | **Explanation** | **Note** | |
| --- | --- | --- | --- | --- |
| **Part I: topological properties for individual nodes** | | | |  |
| Degree |  | is the connection strength between nodes i and j. | The number of direct association interactions for a specific OTU. | |
| Clustering coefficient | $CC_{i}=\frac{2l_{i}}{k_{i}^{'}\left(k_{i}^{'}-1\right)}$ | *l_i_* is the number of links between neighbors of node *i* and *k_i_’* is the number of neighbors of node *i.* | It represents how well a node is connected with its neighbors. | |
| Betweenness | $B_{i}=\sum_{jk} \frac{\sigma(j,i,k)}{\sigma(j,k)}$ | $\sigma(j,k)$is the total number of shortest paths between j and k. | It is used to describe the ratio of paths that pass through the ith node. | |
| **Part II: The overall network topological properties** | | | |  |
| Average connectivity | $avg=\frac{\sum_{i=1}^{n} k_{i}}{n}$ | *k_i_* is degree of node *i* and *n* is the number of nodes. | Higher *avgK* means a more complex network. | |
| Harmonic geodesic distance | $HD={(\frac{1}{n(n-1)}\sum_{i\neq j} d_{ij})}^{-1}$ | *d_ij_* is the shortest path between node *i* and *j*. | A smaller *GD* means that all the nodes in the network are closer. | |
| Average clustering coefficient | $avgCC=\frac{\sum_{i=1}^{n} CC_{i}}{n}$ | *CC_i_* is the clustering coefficient of node *i* | It is used to measure the extent of hierarchical structure present in a network. | |
| Centralization of betweenness | $CB=\sum_{i=1}^{n} (\max\left( B \right)-B_{i})$ | max(*B*) is the maximal value of all betweenness values and *B_i_* represents the betweenness  of ith node. Finally this  value is normalized by the theoretical maximum centralization score. | It is close to 0 for a network in which each node has the same betweenness, and the bigger the more difference among all betweenness values. | |
| Modularity | $Q=\frac{1}{2l}\sum_{i\neq j} \left[A_{ij}-\frac{k_{i}k_{j}}{2l}\right]\delta\left(m_{i},m_{j}\right)$ | *l* is the sum of total links; $A_{ij}$is 1 if node *i* and *j* are connected and 0 otherwise; *k_i_* is the degree of *i*; *m_i_* is the module that *i* belong to; $\delta\left(m_{i},m_{j}\right)$ is 1 if *m_i_*=*m_j_* and 0 otherwise. | It demonstrates how well a network could be naturally divided into modules. | |
